# Development and validation of an intensive care unit acquired weakness prediction model: A cohort study

**DOI:** 10.3389/fmed.2023.1122936

**Published:** 2023-02-22

**Authors:** Zi Yang, Xiaohui Wang, Guangming Chang, Qiuli Cao, Faying Wang, Zeyu Peng, Yuying Fan

**Affiliations:** ^1^Clinical Nursing Teaching Department, The Second Affiliated Hospital of Harbin Medical University, Harbin, China; ^2^School of Nursing, Harbin Medical University, Harbin, China; ^3^Department of Nursing, Shenzhen Qianhai Taikang Hospital, Shenzhen, China; ^4^Office of Medical Ethics Committee, The Second Affiliated Hospital of Harbin Medical University, Harbin, China; ^5^Surgical Laboratory, Department of Medical Education, The First Affiliated Hospital of Jiamusi University, Jiamusi, China

**Keywords:** intensive care unit, intensive care unit acquired weakness, risk prediction, risk factors, model

## Abstract

**Background:**

At present, intensive care unit acquired weakness (ICU-AW) has become an important health care issue. The aim of this study was to develop and validate an ICU-AW prediction model for adult patients in intensive care unit (ICU) to provide a practical tool for early clinical diagnosis.

**Methods:**

An observational cohort study was conducted including 400 adult patients admitted from September 2021 to June 2022 at an ICU with four ward at a medical university affiliated hospital in China. The Medical Research Council (MRC) scale was used to assess bedside muscle strength in ICU patients as a diagnostic basis for ICUAW. Patients were divided into the ICU-AW group and the no ICU-AW group and the clinical data of the two groups were statistically analyzed. A risk prediction model was then developed using binary logistic regression. Sensitivity, specificity, and the area under the curve (AUC) were used to evaluate the predictive ability of the model. The Hosmer-Lemeshow test was used to assess the model fit. The bootstrap method was used for internal verification of the model. In addition, the data of 120 patients in the validation group were selected for external validation of the model.

**Results:**

The prediction model contained five risk factors: gender (OR: 4.31, 95% CI: 1.682–11.042), shock (OR: 3.473, 95% CI: 1.191–10.122), mechanical ventilation time (OR: 1.592, 95% CI: 1.317–1.925), length of ICU stay (OR: 1.085, 95% CI: 1.018–1.156) and age (OR: 1.075, 95% CI: 1.036–1.115). The AUC of this model was 0.904 (95% CI: 0.847–0.961), with sensitivity of 87.5%, specificity of 85.8%, and Youden index of 0.733. The AUC of the model after resampling is 0.889. The model verification results showed that the sensitivity, specificity and accuracy were 71.4, 92.9, and 92.9%, respectively.

**Conclusion:**

An accurate, and readily implementable, risk prediction model for ICU-AW has been developed. This model uses readily obtained variables to predict patient ICU-AW risk. This model provides a tool for early clinical screening for ICU-AW.

## Background

Intensive care unit acquired weakness (ICU-AW) is a common neuromuscular complication in critically ill patients, which manifests mainly as symmetrical weakness of the limbs, decreased reflexes, and muscle atrophy (1). Multiple factors are implicated in the etiology of ICU-AW, such as the duration of mechanical ventilation or length of ICU stay (2–4), and the prevalence varies considerably by patient group. ICU-AW not only leads to increase short- and long-term mortality, but also seriously affects the quality of life of patients (5–7). ICU-AW is an important clinical problem (5), which has become a topic of concern for many scholars locally and internationally. Clinically, ICU-AW is diagnosed by different means, including neuromuscular biopsy and electromyography (8). However, the usefulness of this diagnostic methods is limited in the ICU, so there is no consensus on the gold standard for diagnosis of ICU-AW (9). Many studies (8, 10–13) have attempted to explore the pathogenesis and associated risk factors of ICU-AW over the past few decades, and have greatly contributed to our understanding of the pathophysiology, epidemiological characteristics, and associated risk factors. For a variety of reasons, a small number of patients is still insufficient to assess the independent predictive value of potential risk factors for ICU-AW. There are currently no risk stratification schemes for primary prevention. It has been suggested that quantifying the risk of ICU-AW using a risk prediction model early after admission may be a way to address its delayed diagnosis (14). However, due to different research perspectives, backgrounds and patient populations, the existing ICU-AW risk prediction models differ greatly in the selection of predictors and model efficacy, which to some extent limits the early prediction and early warning management of ICU-AW by clinical medical staff, see Table 1. At present, it is still necessary to establish a risk prediction model suitable for the occurrence of ICU-AW in Chinese patient population (15).

**TABLE 1:** Summary of previous models.

References	Country	Internal validation	External validation	Risk stratification	Limitations
Diaz Ballve et al. (8)	Argentina	No	No	No	Lack of relevant internal and external validation
Wolfe (21)	United States	No	No	No	Lack of internal and external validation
De Jonghe (22)	France	No	No	No	Lack of internal and external validation; the sample size was insufficient
Garnacho-Montero (23)	Spain	No	No	No	The diagnostic tool for ICU-AW was invasive and limited in clinical application; the sample size was insufficient
Hernández-Socorro (24)	Spain	No	No	No	Tools to check for muscle atrophy was expensive and limited in clinical application
Peñuelas (25)	Spain	Yes	No	No	Patients with mild to moderate ICU-AW may be missed and models had not yet been calibrated and validated
Witteveen et al. (26)	Netherlands	Yes	Yes	No	The model was poorly differentiated and required external validation to demonstrate its performance and clinical applicability
Liu (27)	China	No	No	No	Lack of internal and external validation
Wieske et al. (14)	Netherlands	Yes	Yes	No	Differentiation and calibration were poor
Weber-Carstens (28)	Germany	No	No	No	The diagnostic tool for ICU-AW was invasive and limited in clinical application
Miao (29)	China	No	Yes	Yes	Lack of internal validation

Using evidence-based theory, this study aims to use the existing clinical data to predict the occurrence of ICU-AW and develop an early risk prediction model suitable for use by ICU nurses for early detection, dynamic monitoring, and effective prevention.

## Materials and methods

### Design and study participants

This was an observational cohort study. We present the following article in accordance with the TRIPOD reporting checklist. The selected study participants were 400 inpatients in an ICU with four wards in the affiliated hospital of a medical university in Heilongjiang Province from September 2021 to June 2022, and the data of 280 patients collected from September 2021 to March 2022 were used to develop the model. The criteria for inclusion and exclusion of study participants were shown in Table 2.

**TABLE 2:** Criteria for inclusion and exclusion of participants.

Inclusion criteria	Exclusion criteria	Case exclusion criteria
Age ≥ 18 years; The patient was conscious during the bedside muscle strength assessment (awake and responding to at least three of the following simple commands: open or close your eyes, look at me, stick out your tongue, nod your head, and frown); Estimated ICU stay > 24 h; Informed consent of the patient or his/her agent.	Patients with a history of mental illness or cognitive impairment; Patients with neuromuscular diseases such as myasthenia gravis, Guillain-Barré syndrome, or sequelae of stroke before admission to the ICU; Patients who were unable to perform a bedside muscle strength assessment test, such as those with severe trauma; Patients with diseases that could affect motor function or intracranial or spinal cord status abnormalities, such as increased intracranial pressure; Patients admitted to hospital for cardiac arrest or spinal injury; Patients who were expected to die within 48 h.	Patients with incomplete case data who died before the study was completed.

### Sample size

A total of 26 observational variables were included in this study, based on the predictors screened by the research group’s preliminary meta-analysis and expert consultation. According to the modeling sample size formula, the recommended sample size is 5–10 times the number of observed variables (16), considering the limitations of workforce and time in this study, the sample size was set to five times the number of variables. Moreover, the meta-analysis by Zang et al. (17) revealed an incidence of ICU-AW is 51.90%, considering the possible loss of 20% sampled patients, we aimed for a final sample size of ≥300 cases. A total of 400 ICU patients were enrolled actually. According to the logistic regression requirements for development of the model, patients were divided into the modeling group (*n* = 280) and the verification group (*n* = 120) in a ratio of 7:3. In the modeling group, 40 patients (14.29%) had ICU-AW, and 240 patients (85.71%) did not. In the validation group, 21 patients (17.5%) had ICU-AW and 99 patients (82.5%) did not.

### Diagnostic standard

#### ICU-AW

**FIGURE 1:**
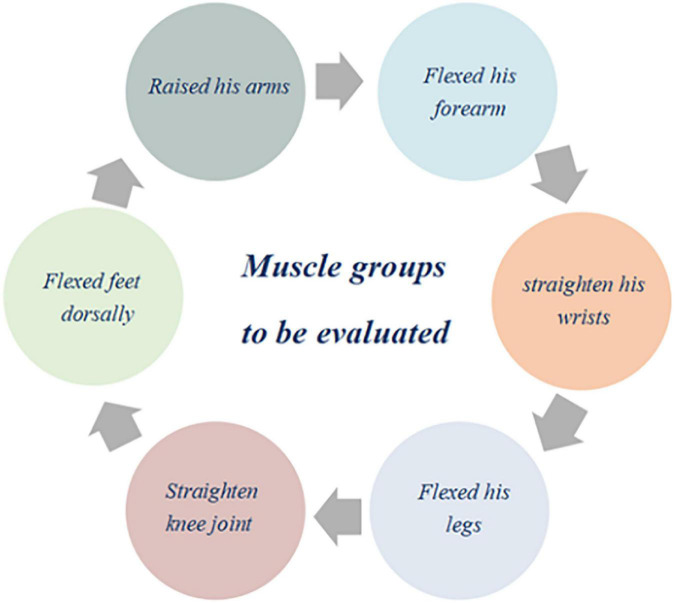
Muscle group sites used for bedside muscle strength assessment.

Intensive care unit acquired weakness bedside muscle strength was assessed using the Medical Research Council (MRC) score recommended by the American Thoracic Society (ATS) in 2014 (18). The sedative infusion was discontinued at least 30 min before using the MRC scale, which required the patient to be awake and able to response to at least three of the following simple commands: open or close your eyes, look at me, stick out your tongue, nod your head, or frown. After these commands were performed, muscle strength was assessed using the MRC scale. The MRC scale includes six pairs of muscle mass grading, each with a score of 0 to 5 points; the left and right sides are assessed simultaneously (Figure 1). The total score was 0–60, with a total score less than 48 as the basis for diagnosing of ICU-AW. The overall Cronbach α coefficient of the MRC scale was 0.912. The evaluation criteria of the scale are shown in Table 3.

**TABLE 3:** Bedside muscle strength assessment criteria.

Score	Symptoms
0	No significant muscle contractions
1	Although there are muscle contractions, there is no limb movement
2	Although the limbs can be moved, they cannot resist gravity
3	Limbs can not only move, but also resist gravity
4	Limbs can resist gravity and resist a certain degree of resistance
5	Normal muscle strength

#### Systemic inflammatory response syndrome (SIRS)

According to the 1991 Joint Meeting of the American College of Chest Physicians and the Critical Care Society for the diagnosis of SIRS, SIRS can be diagnosed if two or more of the following criteria are met: (1) body temperature >38^°^C or <36^°^C; (2) heart rate >90/min; (3) respiration >20/min or hyperventilation, arterial partial pressure of carbon dioxide (PaCO_2_) <32 mmHg (1 mmHg = 0.133 kPa); (4) White blood cell count >12 × 10^9^/L, or <4 × 10^9^/L, or immature granulocytes >10%.

#### Acute kidney injury

According to the KDIGO Clinical Practice Guidelines for Acute Kidney Injury published by Kidney Disease: Improving Global Outcomes (KDIGO) (19) acute kidney injury can be diagnosed if one of the following criteria is met:(1) increased serum creatinine ≥0.3 mg/mL (≥26.5 μmol/L) within 48 h; (2) increased serum creatinine more than 1.5 times baseline within the past 7 days; (3) Urine volume within 6 h ≤0.5 ml/(kg⋅h).

#### Delirium

Delirium assessments were performed twice daily using the Confusion Assessment Method for the Intensive Care Unit (CAM-ICU), increasing the number of assessments as necessary (20).

### Data collection

The questionnaire was designed that included 26 risk factors. The results of our previous meta-analysis were included, and we consulted the opinions of experts in the ICU of our research center (including physicians and nurses with >10 years’ experience who were engaged in ICU-AW related research). Based on the preliminary meta-analysis results and expert opinions, we finally made this questionnaire for researchers to collect data. Patient datasets were collected through the Hospital Information System (HIS) and included data on a total of 26 risk factors in the following four categories see Table 4. All patient data were entered and reviewed by the researcher using Excel software. For data collection, all the researchers were trained uniformly to ensure consistency. The researchers participated in the entire data collection process and recorded it. The included participants were assessed once daily by a critical care nurse and a researcher using the MRC scale in an awake state. If the patient develops ICU-AW, the evaluation is discontinued; If the patient did not develop ICU-AW, evaluation was continued on day 2 by another investigator until the patient was transferred out of the ICU. After the study began, the researchers summarized the data collected each week and provided timely feedback and adjustments to problems that occurred during the data collection process.

**TABLE 4:** Patients clinical data.

General information	Age, gender, BMI, history of alcohol consumption, history of underlying medical conditions
Disease factors	APACHE II score, SOFA score, SIRS, shock, acute kidney injury, delirium, infectious disease
Therapeutic factors	Length of ICU stay, days of immobilization, mechanical ventilation time, CRRT (application time > 24 h), parenteral nutrition (application time > 24 h), norepinephrine (application time > 24 h), glucocorticoids (application time > 24 h), sedative/analgesic drugs (application time > 24 h), neuromuscular blockers (application time > 24 h), aminoglycoside antibiotics (application time > 24 h)
Laboratory data	Blood glucose (blood glucose Q8 h), lactic acid level, serum albumin concentration, ion calcium concentration

BMI, body mass index; APACHE II, acute physiology and chronic health evaluation; SOFA, sequential organ failure assessment; SIRS, systemic inflammatory response syndrome; CRRT, continuous renal replacement therapy.

### Statistical analysis

IBM SPSS Statistics version 27.0 and MedCalc software were used for statistical analysis. Nominal variables are reported as numbers and proportions (%), and the chi-square test was used for group comparisons. Continuous data with a normal distribution are reported as the mean ± standard deviation (mean ± SD), and a *t*-test was used for group comparisons. Continuous data with a non-normal distribution are reported as medians and interquartile range M (*P*_25_, *P*_75_), and the non-parametric Wilcoxon rank sum test was used for group comparisons.

To develop the model, we tested the correlations between predictors (*P* < 0.05 in the univariate analysis) and ICU-AW using a binary logistic regression model (using step forward regression). The model was corrected using the Hosmer-Lemeshow goodness of fit test (*P* > 0.05 indicated good model fit), and the discriminatory capacity of the model was indicated by the receiver operating characteristic (ROC) area under the curve (AUC). After the Youden index was used to determine the optimal critical value of the ROC curve, the sensitivity and specificity of the prediction model were estimated. The model was internally validated using the bootstrap method with 500 replications. A normality test was conducted according to the scores of patients in the model validation group and the risk levels were categorized as follows: <50% (low risk), 50–75% (medium risk), and >75% (high risk).

## Results

**FIGURE 2:**
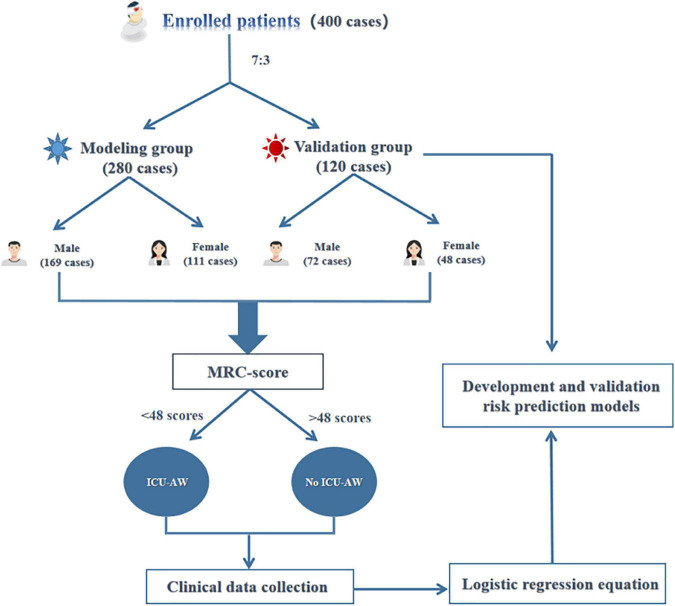
Research flow chart.

The 280 patients in the modeling group of this study included 169 males (60.36%) and 111 females (39.64%); age: 59.41 ± 14.96 years. ICU-AW occurred in 40 patients, including 16 males (40%) and 24 females (60%), and the incidence of ICU-AW was 14.29%. The research flow chart is shown in Figure 2.

### Univariate analysis of ICU-AW in ICU patients

The study population was divided into the ICU-AW group and no ICU-AW groups and univariate analysis of the clinical data of the two groups was undertaken. The results showed that there were statistically significant differences between the two groups for age, gender, body mass index, shock, length of ICU stay, use of norepinephrine, days of immobility, parenteral nutrition, mechanical ventilation time, and infectious diseases (*P* < 0.05) (Table 5).

**TABLE 5:** Univariate analysis of intensive care unit acquired weakness (ICU-AW) risk factors.

Categories	ICU-AW (*n* = 40)	No ICU-AW (*n* = 280)	χ^2^/Z value	*P*-value
Age, mean ± SD	67.33 ± 12.21	58.09 ± 14.99	–3.70	<0.001
**Gender, *n* (%)**				
Male	16	153	8.08	0.01
Female	24	87		
BMI, M (*P*_25_, *P*_75_), kg/m^2^	25.00 (23.30, 27.33)	24.10 (22.23, 25.80)	–2.72	0.01
**History of alcohol consumption, *n* (%)**				
Yes	3	28	0.61	0.44
No	37	212		
**History of underlying medical conditions, *n* (%)**				
Yes	29	145		
No	11	95	2.13	0.15
APACHE II score, M (*P*_25_, *P*_75_),	16.00 (12.25, 20.75)	15.00 (11.00, 19.00)	–1.20	0.23
SOFA score, M (*P*_25_, *P*_75_)	3.00 (2.00, 4.00)	2.00 (1.00, 4.00)	–1.10	0.27
**SIRS, *n* (%)**				
Yes	11	56	0.33	0.57
No	29	184		
**Shock, *n* (%)**				
Yes	12	37	5.05	0.03
No	28	203		
**Acute kidney injury, *n* (%)**				
Yes	5	32	0.02	0.89
No	35	208		
**Delirium, *n* (%)**				
Yes	3	36	1.61	0.21
No	37	204		
Length of ICU stay, M (*P*_25_, *P*_75_), day	8.00 (5.00, 13.75)	2.00 (1.00, 4.75)	–7.11	<0.001
Days of immobilization M (*P*_25_, *P*_75_), day	6.00 (4.00, 8.00)	1.00 (0.00, 3.00)	–7.88	<0.001
Mechanical ventilation time M (*P*_25_, *P*_75_), day	4.00 (3.00, 7.00)	1.00 (0.00, 2.00)	–6.72	<0.001
**CRRT, *n* (%)**				
Yes	9	50	0.06	0.81
No	31	190		
**Norepinephrine, *n* (%)**				
Yes	19	69	5.59	0.02
No	21	171		
**Glucocorticoids, *n* (%)**				
Yes	8	35	0.77	0.38
No	32	205		
**Sedative/Analgesic drugs, *n* (%)**				
Yes	20	122	0.01	0.92
No	20	118		
**Neuromuscular blockers, *n* (%)**				
Yes	5	15	2.02	0.16
No	35	225		
**Parenteral nutrition, *n* (%)**				
Yes	4	59	4.18	0.04
No	36	181		
Blood glucose, M (*P*_25_, *P*_75_), mmol/L	9.35 (6.75, 12.25)	8.45 (6.60, 11.68)	–1.16	0.25
Lactic acid level, M (*P*_25_, *P*_75_), mmol/L	1.90 (1.20, 2.75)	1.75 (1.10, 3.20)	–0.54	0.59
Serum albumin concentration M (*P*_25_, *P*_75_), g/L	33.70 (29.03, 40.58)	33.20 (28.73, 38.50)	–0.91	0.36
Ion calcium concentration mean ± SD, mmol/L	1.90 ± 0.45	1.90 ± 0.46	–0.06	0.96
**Infectious disease, *n* (%)**				
Yes	24	92	6.63	0.01
No	16	148		
**Aminoglycoside antibiotics, *n* (%)**				
Yes	8	56	0.2	0.64
No	32	184		

### Binomial logistic analysis of ICU-AW in patients with ICU

**FIGURE 3:**
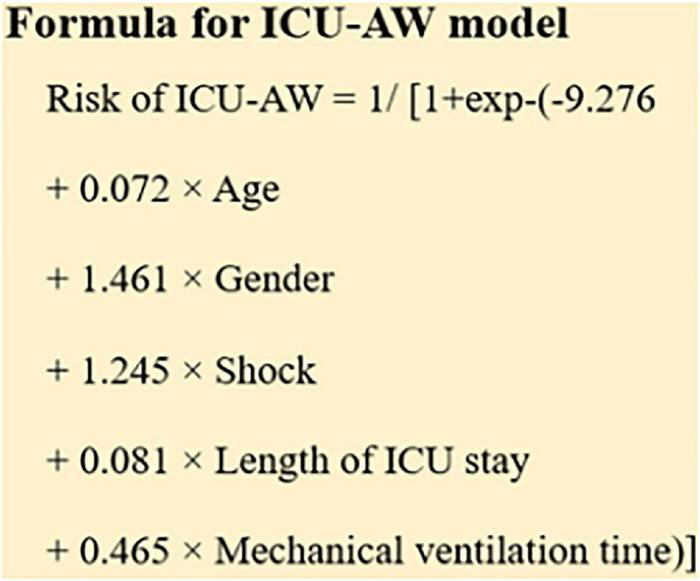
Prediction model formula.

The results are shown in Tables 6, 7. The results showed that gender (OR: 4.31, 95% CI: 1.682–11.042), shock (OR: 3.473, 95% CI: 1.191–10.122), mechanical ventilation time (OR: 1.592, 95% CI: 1.317–1.925), length of ICU stay (OR: 1.085, 95% CI: 1.018–1.156) and age (OR: 1.075, 95% CI: 1.036–1.115) were independent risk factors for ICU-AW in ICU patients. The prediction model formula is shown in Figure 3.

**TABLE 6:** Independent variable assignment table.

Independent variables	Assignment method
Age (years) Mechanical ventilation time, day Length of ICU stay, day Days of immobilization, day BMI, kg/m^2^ Gender	Enter the original value Enter the original value Enter the original value Enter the original value Enter the original value Male = 0; Female = 1
Shock	No = 0; Yes = 1
Norepinephrine	No = 0; Yes = 1
Parenteral nutrition	No = 0; Yes = 1
Infectious disease	No = 0; Yes = 1

**TABLE 7:** Binomial logistic regression analysis of intensive care unit acquired weakness (ICU-AW) risk factors.

Independent variables	Regression coefficient	Standard error	Wald value	*P*-value	OR value	95% CI
Age	0.072	0.019	14.477	<0.001	1.075	1.036∼1.115
Gender	1.461	0.480	9.260	0.002	4.310	1.682∼11.042
Shock	1.245	0.546	5.202	0.023	3.473	1.191∼10.122
Length of ICU stay	0.081	0.032	6.342	0.012	1.085	1.018∼1.156
Mechanical ventilation	0.465	0.097	23.123	<0.001	1.592	1.317∼1.925
Constant	–9.276	1.508	37.844	<0.001	–	–

### The fitting and effect analysis of ICU-AW risk prediction model

**FIGURE 4:**
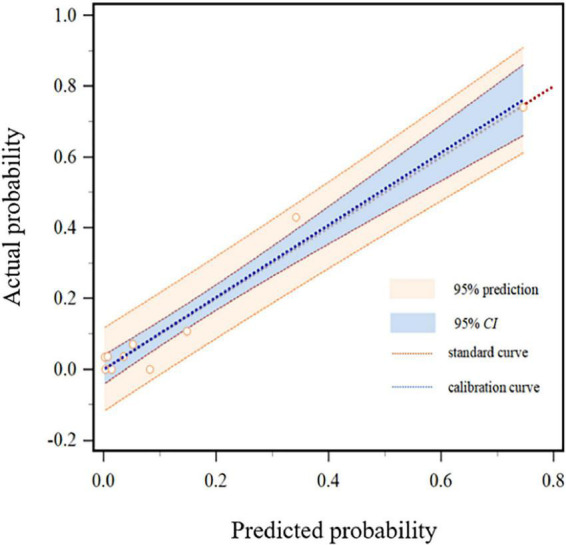
Calibration degree of the logistic regression model.

**FIGURE 5:**
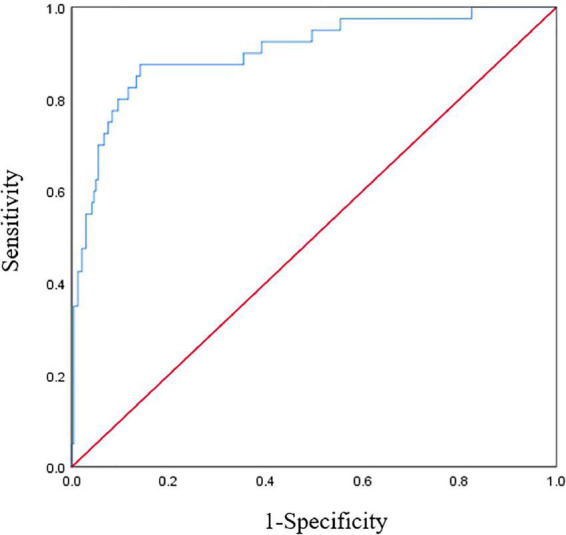
Receiver operating characteristic (ROC) curve of the intensive care unit acquired weakness (ICU-AW) risk prediction model.

**FIGURE 6:**
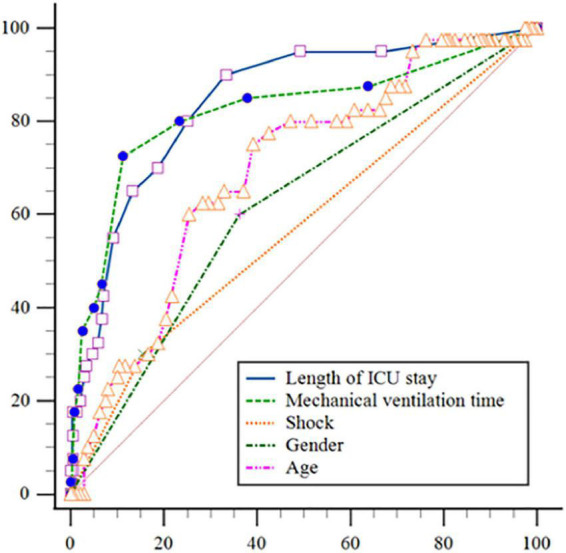
Receiver operating characteristic (ROC) curves of each variable in the intensive care unit acquired weakness (ICU-AW) risk prediction model.

The Hosmer-Lemeshow test was χ*^2^* = 7.656 and the degree of freedom was eight, *P* = 0.468. A Calibration curve as shown in Figure 4. The ROC curve of the ICU-AW risk prediction model as shown in Figure 5. The area under the ROC curve of this model was 0.904, 95% *CI* (0.847, 0.961), the Youden index was 0.733, the optimal truncation value was 0.145, the sensitivity was 87.5%, and the specificity was 85.8%. See Figure 6, Table 8 for the area under the curve of each predictive variable in the model.

**TABLE 8:** The area under the curve of each predictive variable in the mode.

Variable	AUC	SE^a^	95% CI^b^
Age	0.694	0.0432	0.637∼0.748
Gender	0.619	0.0422	0.559∼0.676
Shock	0.573	0.0385	0.513∼0.632
Length of ICU stay	0.845	0.0333	0.798∼0.886
Mechanical ventilation time	0.823	0.0432	0.773∼0.866

^a^DeLong et al., 1988; ^b^Binomial exact.

### Clinical validation and risk classification of the ICU-AW risk prediction model

The 0.632 Bootstrap was used to re-sample for 500 times, and the AUC was 0.889. A total of 120 patients admitted to the ICU in the same hospital from March to May 2022 were selected as the model validation group. There were 72 males and 48 females. The age was 60.02 ± 15.33 years. According to the formula of this prediction model, ICU-AW was considered to occur when Z ≥ 0.145. ICU-AW occurred in 21 cases (17.5%). The sensitivity of the model was 71.4% (15 cases, with six cases misjudged). There were 99 cases without ICU-AW; 92 cases were predicted by the model, seven cases were misjudged, and the specificity was 92.9%. The overall accuracy of the model was 89.2%.

Normality tests were conducted according to the scores of patients in the model validation group, and the results showed a skewed distribution, *P*_25_ = –4.42, *P*_50_ = –3.30, *P*_75_ = –1.61. The risk levels were divided as follows: <50% (predicted probability value <–3.30) was considered low risk; 50–75% (predicted probability value –3.30 to 1.61) was considered medium risk; and >75% (predicted probability value >–1.61) was considered high risk. Of the 120 patients in the validation group, 59 patients (49.2%) were classified into the low-risk group. There were 31 cases in the moderate risk group, accounting for 25.8%, and 30 cases in the high-risk group, accounting for 25%. A model score <–3.303 was defined as a low-risk group, and the goal of the health care provider was to reduce exposure to ICU-AW risk factors. A model score of >–3.303 and <–1.606 defined the medium-risk group, this group required closer monitoring, with a focus on related risk factors. A model score of >–1.606 was considered to indicate high-risk, and necessary measures were required to prevent the occurrence of ICU-AW in patients in this group.

## Discussion

In this cohort study, we developed and validated a model that reliably predicted the likelihood of ICU-AW based on five risk factors: gender, age, length of ICU stay, mechanical ventilation time, and shock. The model was found to have great discrimination and calibration, which can provide a reference for the early clinical identification of ICU-AW.

### Independent predictive factor analysis of ICU-AW

As expected, the age of ICU-AW patients in this study was significantly higher than that of the no ICU-AW patients, suggesting that age is an independent risk factor for ICU-AW. As many countries around the world gradually enter an aging society, the average age of ICU patients increases, and patients will have problems such as decreased muscle reserve and muscle strength before they are admitted to ICU. However, the severity of the disease accelerates these changes, and even leads to the onset of muscle decay syndrome (30, 31), which may be the mechanism by which age influences the occurrence of ICU-AW. Related studies included age as one of the independent predictors of ICU-AW in their risk prediction models (8, 14), which is consistent with our study. In addition, a significant association was observed between gender and ICU-AW (OR: 4.310, 95% CI: 1.682–11.042). Through independent variable assignment, we found that the risk of ICU-AW in women was up to 4.31 times higher than that in men, which was consistent with other studies (18, 26, 32, 33). Nevertheless, whether gender is an exact independent risk factor for ICU-AW remains controversial, and the mechanism is unclear. Although an observational study did not find an association between sex hormones and ICU-AW (34), for the purpose of risk prevention and control, clinical staff should also regard women as a high-risk group for ICU-AW and focus on monitoring. Identifying immutable factors (e.g., age, sex) is crucial for early prediction and to understand the causal relationship between the relevant variables and ICU-AW.

Interestingly, shock was strongly associated with the development of ICU-AW as an independent predictor, which is related to the possibility that microcirculation disorders may lead to neuronal damage and axis mutations. A prospective study conducted by Anastasopoulos et al. (31) confirmed that shock is an important risk factor for ICU-AW in critically ill patients, which is consistent with the results of this study. However, few studies have reported the association between shock and ICU-AW in recent years. This lack of information is surprising because shock is not uncommon in ICU patients. This predictor (shock) is an important finding in understanding factors associated with ICU-AW.

Our study found that the duration of mechanical ventilation was positively correlated with ICU-AW, that is, the longer the duration of mechanical ventilation, the higher the incidence of ICU-AW. This finding is consistent with the results from various studies (10–12, 35, 36). Long-term mechanical ventilation can cause certain pathophysiological changes in the diaphragm, resulting in atrophy and dysfunction of the diaphragm. Within 48 h of mechanical ventilation, diaphragm injury was positively correlated with ventilation support, and diaphragm loss was most obvious in volumetric respiratory support mode (37). Ventilator-induced diaphragm dysfunction may also be associated with ICU-AW. We observed a significant correlation between the length of ICU stay and ICU-AW. Patients in ICU-AW group had spent significantly longer in ICU than those without ICU AW, which was consistent with the findings of Hermans and Van den Berghe (38). This may be due to the fact that ICU patients are prone to microcirculation disorders and malnutrition, leading to decreased muscle strength, and patients may show anxiety and depression during ICU treatment, which affects compliance with early rehabilitation. The risk of ICU-AW increases with the length of ICU stay. Although there is a lack of direct evidence that psychological factors are independent risk factors for ICU-AW, we hypothesized that psychological factors may influence patient compliance with early rehabilitation and exercise, leading to the occurrence of ICU-AW. This gives us an important inspiration that ICU medical staff should not neglect the psychological care of patients while providing specialized care, which is of inestimable value in improving the clinical outcome and reducing the occurrence of post-ICU syndrome. It also provides a new idea for further verifying the relationship between psychological factors and ICU-AW.

Researchers believe that immobilization is the primary risk factor for ICU-AW (13). To our surprise, days of immobilization were found to be strongly associated with ICU-AW in univariate analysis believe that immobilization is the primary risk factor for ICU-AW (13). To our surprise, days of immobilization were found to be strongly associated with ICU-AW in univariate analysis (*P* < 0.001), but in logistic regression analysis, the number of days of immobilization did not appear to be an independent risk factor for ICU-AW and was not included in the model. This may be related to confounding factors and individual patient differences. Adding this factor (immobilization) to the model reduced its sensitivity to other covariables, so we did not include it in this model. Even so, ICU medical staff should carry out scientific and reasonable treatment and nursing and strictly manage the duration of physical restraint to reduce the risk of ICU-AW.

### The use of the risk prediction model

This model can predict the risk of ICU-AW at the beginning of admission to the ICU. After admission to ICU, the patient’s indicators were put into the model formula (Z = 0.081 × length of stay in ICU + 0.465 × days of mechanical ventilation + 1.245 × shock + 1.461 × gender + 0.072 × age-9.276). Patients were classified as low risk when model score <–3.303; When –3.303 <model scores <–1.606, it was classified as medium risk. Medical staff should pay enough attention to it and focus on monitoring various indicators to avoid the occurrence of ICU-AW in patients; When the model score is >–1.606, which is classified as high risk. Patients who have a higher probability of developing ICU-AW, and medical staff should pay high attention to it and actively intervene in patients.

### Advantages and limitations

Our study had certain advantages. First, the cohort study design is helpful to improve the universality of the risk prediction model (39), since it allowed us to carefully measure and record predictors and outcomes to improve their applicability to ICU patients. Second, in the process of data collection, unified diagnostic criteria were used to evaluate the occurrence of ICU-AW, with blind judgment of outcome indicators to avoid participative bias as much as possible. At the same time, the risk prediction model developed in this study was evidence-based to screen the related risk factors of ICU-AW. The included risk factors were scientific, comprehensive, and in line with clinical requirements. In this risk prediction model developed by logistic regression, although it is difficult to completely avoid the influence of confounding factors, we tried to reduce the influence of confounding factors as much as possible by explaining all major variables. Finally, the predictive variables included in the model are easy to measure, data acquisition is convenient, and clinical indicators can be obtained at an early stage of ICU admission, enabling ICU medical staff to identify the risk of ICU-AW as early as possible. This risk prediction model developed in this study has great performance and high predictive value, which is helpful for medical staff to implement scientific prevention strategies according to the predictive factors, to reduce the incidence of ICU-AW.

However, our study has some limitations. Firstly, due to the strict limitations of the inclusion criteria (requiring patients to be conscious), while the use of MRC scale is helpful for bedside muscle strength assessment, it may miss patients who are unconscious but have ICU-AW. Therefore, we believe that the incidence of ICU-AW may be underestimated (14.39% in the model group and 17.5% in the validation group in this study). The clinical assessment of ICU-AW is challenging, and one of the most critical factors limiting its assessment is altered patient consciousness, which can be due to a variety of reasons, including sedative drug use. We tried to overcome this problem by suspending sedative use prior to bedside muscular strength assessment. However, as other methods for diagnosing ICU-AW are invasive and expensive, it is necessary to use the MRC scale for bedside muscle strength assessment in clinical practice. Secondly, the risk prediction model constructed in this study is a static model, which can calculate the probability of ICU-AW after admission to ICU. However, since patients’ health may improve or deteriorate at any time during their stay in ICU, the probability of developing ICU-AW may also change; our model lacks consideration for changes in patients’ health status. Most risk prediction models for ICU-AW have this limitation, but even so, it makes sense to develop a risk prediction model that uses dynamic parameters as predictors, such as APACHE II scores. New risk factors for ICU-AW may emerge during ICU treatment, possibly during or after ICU admission, suggesting that the model needs to be updated in the future. Of course, this also provides an opportunity to further improve the performance of the model. Thirdly, COVID-19 patients were not taken into account in this risk prediction model. This is because COVID-19 patients were not treated in this research center but uniformly transferred to other designated hospitals. Therefore, no COVID-19 patients were collected during the data collection process. However, it also provides a new perspective for subsequent studies to investigate the association between COVID-19 patients and the development of ICU-AW. Fourthly, it is of great significance to establish a risk prediction model for predicting severe ICU-AW. Due to the insufficient sample size of patients with severe ICU-AW in this study, a prediction model suitable for predicting severe ICU-AW patients was not established, and the sample size will be further increased in the future. Finally, both the development and validation of the model were carried out in one hospital, and the clinical applicability of the results of this study in other countries or regions needs further evaluation. Large sample data are still needed to verify the model in the future, but the process of model validation is also the process of model optimization.

## Conclusion

This model developed in this study can predict the occurrence of ICU-AW in patients admitted to ICU, which will help health care workers target preventive measures. Although this study illustrates associations between certain variables and outcome indicators, further research is needed to ascertain whether these variables have a causal relationship. Rather than developing new models, we recommend updating predictive models derived from existing clinical data and conducting further confirmatory studies. Establishing the risk prevention and control mechanism and improving the risk prevention and control consciousness of ICU medical staff are of irreplaceable value for improving the clinical outcomes of patients.

## Data availability statement

The raw data supporting the conclusions of this article will be made available by the authors, without undue reservation.

## Ethics statement

The studies involving human participants were reviewed and approved by the Ethics Committee of The Second Affiliated Hospital of Harbin Medical University. The patients/participants provided their written informed consent to participate in this study.

## Author contributions

YF: conception and design and administrative support. ZY, XW, and GC: provision of study materials or patients. ZY, XW, GC, and QC: collection and assembly of data, data analysis, and interpretation. All authors contributed to the manuscript writing and approved the submitted version.
